# String Phase in an Artificial Spin Ice

**DOI:** 10.1038/s41467-021-26734-6

**Published:** 2021-11-11

**Authors:** Xiaoyu Zhang, Ayhan Duzgun, Yuyang Lao, Shayaan Subzwari, Nicholas S. Bingham, Joseph Sklenar, Hilal Saglam, Justin Ramberger, Joseph T. Batley, Justin D. Watts, Daniel Bromley, Rajesh V. Chopdekar, Liam O’Brien, Chris Leighton, Cristiano Nisoli, Peter Schiffer

**Affiliations:** 1 Department of Applied Physics, Yale University, New Haven, CT 06511 USA; 2 Department of Physics, University of Illinois at Urbana-Champaign, Urbana, IL 61801 USA; 3 Frederick Seitz Materials Research Laboratory, University of Illinois at Urbana-Champaign, Urbana, IL 61801 USA; 4 Theoretical Division and Center for Nonlinear Studies, MS B258, Los Alamos National Laboratory, Los Alamos, NM 87545 USA; 5 Department of Physics and Astronomy, Wayne State University, Detroit, MI 48201 USA; 6 Department of Chemical Engineering and Materials Science, University of Minnesota, Minneapolis, MN 55455 USA; 7 School of Physics and Astronomy, University of Minnesota, Minneapolis, MN 55455 USA; 8 Department of Physics, University of Liverpool, Liverpool, L69 3BX United Kingdom; 9 Advanced Light Source, Lawrence Berkeley National Laboratory, Berkeley, CA 94720 USA; 10 Department of Physics, Yale University, New Haven, CT 06511 USA

**Keywords:** Topological defects, Magnetic properties and materials, Ferromagnetism

## Abstract

One-dimensional strings of local excitations are a fascinating feature of the physical behavior of strongly correlated topological quantum matter. Here we study strings of local excitations in a *classical* system of interacting nanomagnets, the Santa Fe Ice geometry of artificial spin ice. We measured the moment configuration of the nanomagnets, both after annealing near the ferromagnetic Curie point and in a thermally dynamic state. While the Santa Fe Ice lattice structure is complex, we demonstrate that its disordered magnetic state is naturally described within a framework of emergent strings. We show experimentally that the string length follows a simple Boltzmann distribution with an energy scale that is associated with the system’s magnetic interactions and is consistent with theoretical predictions. The results demonstrate that string descriptions and associated topological characteristics are not unique to quantum models but can also provide a simplifying description of complex classical systems with non-trivial frustration.

## Abstract (web summary)

Strings of local excitations are interesting features of a strongly correlated topological quantum matter. Here, the authors show that Boltzmann-distributed strings of local excitations also describe the topological physics of the Santa Fe geometry of artificial spin ice, which is a classical thermal system.

## Introduction

Numerous exotic phenomena arise in strongly correlated many-body systems, even when the underlying interactions are simple, and artificial spin ice arrays composed of coupled single-domain nanomagnets are an important class of such systems^1–3^. While artificial spin ice studies originally focused on connections to the frustration-induced phenomena seen in pyrochlore spin ice materials^4^, such as monopole-like excitations^5^, the field has now expanded to include a wide range of exotic behavior in carefully designed geometries^6–16^. We have experimentally studied the Santa Fe Ice geometry of artificial spin ice^17,18^, demonstrating that the local excitations among the nanomagnet moments are correlated in Boltzmann-distributed one-dimensional strings. One-dimensional strings of local excitations are an important characteristic in strongly correlated topological quantum matter^19–24^, and our data demonstrate that such strings can also be observed in a classical thermal system.

## Results

**Fig. 1:**
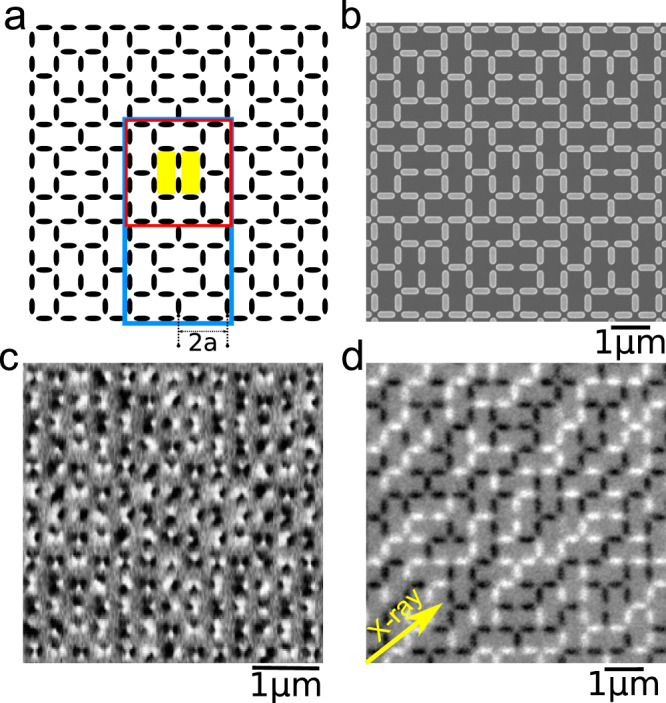
Santa Fe Ice. **a** Schematic of the Santa Fe Ice (SFI) geometry, where each element represents a single-domain nanomagnet, and the lattice constant is *a*. The unit cell (indicated in blue) is made of two composite squares (one of which is indicated in red). Each composite square has eight rectangular plaquettes, which can be categorized as pairs of “interior” plaquettes (indicated by yellow shading) that are separated by a pair of islands, and “peripheral” plaquettes that surround them. **b** Scanning Electron Microscopy (SEM) image of SFI. **c** Magnetic force microscopy (MFM) image of SFI, in which each ferromagnetic island has black and white contrasting ends, indicating the moment poles. **d** X-ray magnetic circular dichroism photoemission electron microscopy (XMCD-PEEM) image of SFI, in which the entire islands are either black or white, indicating the magnetic moment direction through its component projected onto the incident X-ray beam (yellow arrow).

The structure of Santa Fe Ice (SFI)^17,18^ is shown in Fig. 1a, where each island is a single-domain nanoscale ferromagnet that behaves like a binary Ising-like moment. Note that the structure of SFI, while somewhat complex at first sight, is obtained in a straightforward manner by removal of a subset of moments from the simple square ice lattice^17^. Figure 1b shows a scanning electron microscope image of one of our experimental samples, and Fig. 1c, d show experimental measurements of the individual moments in SFI, as described in detail below. The unit cell of SFI, indicated in Fig. 1a, is composed of two composite large squares, each composed of eight elementary rectangular plaquettes consisting of six moments—two interior plaquettes (shaded in yellow) and six peripheral plaquettes (unshaded).

**Fig. 2:**
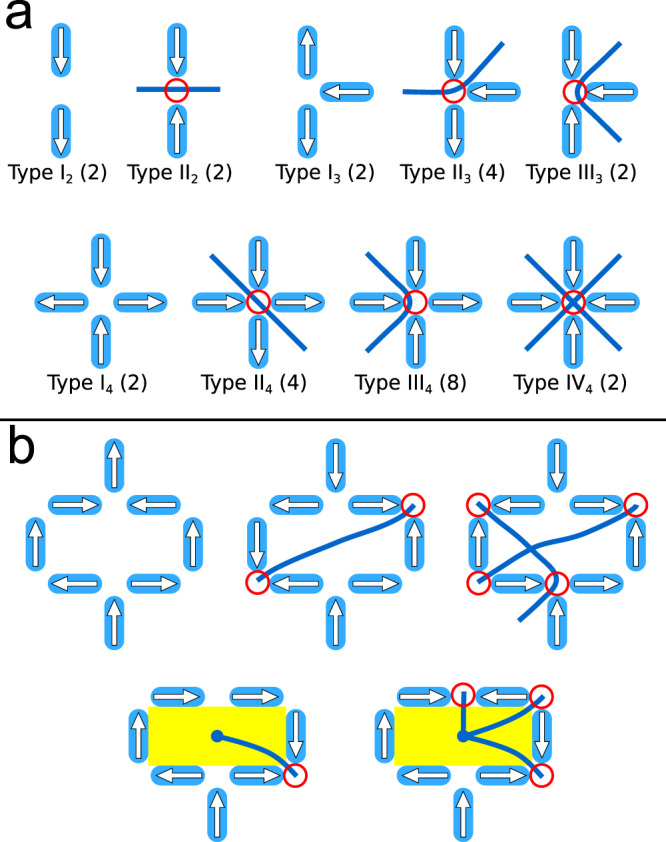
Vertex types and string representation. **a** Vertex moment configurations for different vertex coordination numbers, *z* = 2, 3, and 4, arranged in order of increasing energy, where the arrows indicate moment direction and each red circle denotes an excited state of the vertex (i.e., an unhappy vertex). The numbers in parentheses show the degeneracy for each given vertex type. The blue lines represent string segments “connecting” the plaquettes through the unhappy vertices. **b** Examples of moment and string configurations for peripheral (unshaded) and interior (yellow-shaded) plaquettes. Because of the SFI lattice geometry, a peripheral plaquette is connected by an even number of unhappy vertices, and an interior plaquette is connected by an odd number.

We first analyze the structure of SFI to establish a framework for the analysis of our experimental data. Following previous analyses of artificial spin ice systems^1–3^, we describe SFI in a near-neighbor approximation via a vertex model, i.e., we consider the system through the states of the lattice vertices, as defined by the possible configurations of moments at each vertex of the lattice (Fig. 2a). SFI belongs to the class of artificial spin ices that are “vertex frustrated”^17,18^. Rather than the individual magnetic moments being frustrated in their interactions, as is typical for geometrically frustrated magnets, frustration arises in the spatial allocation of low-energy vertex configurations. In other words, the moments cannot be arranged such that each vertex is in its local ground state (the ground state for the vertex moments when considering just the interactions within the vertex), because the lattice structure of SFI forces some fraction of the vertices to be in a local excited state. These excited vertices have been dubbed “unhappy vertices” in previous works^17,25^, contrasting with “happy vertices” that are in their local ground states.

Within this vertex framework, the proper description of the disorder in SFI is not in terms of magnetic moments but rather in terms of the degenerate allocation of unhappy vertices and of the rules controlling their constrained disorder. In Fig. 2a, we show all possible moment configurations on each vertex type, noting that vertices in this lattice can include either two, three, or four moments, i.e., they can have a coordination number of *z* = 2, 3, or 4. We describe two plaquettes bordered by an unhappy vertex as being “connected” with each other by the unhappy vertex, as illustrated in Fig. 2b where the connected plaquettes are each joined by a blue line drawn through the pair of moments whose relative configuration differs from that in a vertex ground state^8,26^.

Since the two moments in the center of each composite square form a *z* = 2 vertex, within the particular structure of SFI, each of the interior plaquettes must be connected by an *odd* number of unhappy vertices around its edges. Those plaquettes are therefore intrinsically frustrated in that they cannot have all vertices in a ground state. Similarly, each of the peripheral plaquettes must be connected by an *even* number of unhappy vertices (including possibly zero). These two conditions, illustrated and explained in detail in Supplementary Note 2, can be considered as topological constraints: there must be at least one unhappy vertex connected with each interior plaquette, and there could be zero or any even number connected with each peripheral plaquette.

The consequences of viewing SFI through the connected plaquettes are striking. Consider an unhappy vertex that connects an interior plaquette to a peripheral plaquette. In order to yield an even number of unhappy vertices for that peripheral plaquette, a second unhappy vertex must also be connected to it. That vertex in turn connects to another peripheral plaquette, and it is thus natural to attach the unhappy vertices together and visualize their connectivity as one-dimensional strings within the lattice^18^. If we put line segments through each unhappy vertex connecting those plaquettes, as in Fig. 2, and then attach the segments, we find that all the unhappy vertices can be represented as belonging to such strings, with the string length measured as the number of connected unhappy vertices. These strings typically only start and end in the interior plaquettes, because of the odd number of connecting vertices in those plaquettes, although loops that only include peripheral plaquettes are possible at higher temperatures (see Supplementary Fig. 3).

Since the strings represent topological constraints of the SFI moment ensemble, this string representation is valid at any temperature, but it is particularly useful in considering low-energy states of the system. In fact, Monte Carlo simulations demonstrate that the two possible ground states of the moment configuration are characterized by their string configurations (see Supplementary Note 1). For larger next-nearest-neighbor interactions relative to nearest-neighbor interactions, the ground state is long-range-ordered, with strings of length one connecting pairs of contiguous interior plaquettes. In the limit of larger nearest-neighbor interactions, the ground state is characterized by a disordered and highly degenerate state of strings connecting separated interior plaquettes. Our experiments probe this latter regime, as demonstrated in the data below.

We now turn to the experimental behavior of SFI, as viewed within the framework of unhappy vertices being considered collectively as correlated strings. We studied permalloy (Ni_80_Fe_20_) SFI both in a *static* configuration after high temperature annealing and in a *dynamic* state undergoing thermally induced moment reversals. In these two distinct cases, which correspond to thicker and thinner permalloy islands, we image the moment configurations through magnetic force microscopy (MFM) and x-ray magnetic circular dichroism photoemission electron microscopy (XMCD-PEEM), respectively. Details of island size, lattice constant, sample fabrication, and measurement protocols are given in the Methods section, and representative images of the moment configurations obtained with each technique are shown in Fig. 1c, d. In both cases we characterize the fractions of the different vertex states and we extract the distribution of string lengths, *L*, using graph-theoretical techniques described in Supplementary Note 5.

**Fig. 3:**
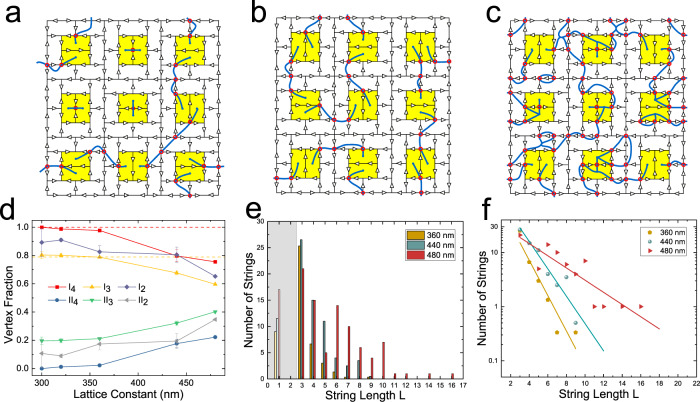
Thermal annealing experimental results. **a–c** Examples of moment configurations for small portions of the arrays measured after annealing, with both the moments and strings represented for samples with lattice constants of  360 nm, 320 nm, and 480 nm, respectively. Additional raw MFM data are given in Supplementary Note 3. **d** The vertex fractions observed after annealing as a function of lattice constant (error bars represent standard deviations of the data). With decreasing lattice constant, i.e., increasing interactions, the fractions converge to those expected for the disordered string ground state. Dashed lines are the expected vertex fraction of I_4_ and I_3_ for the disordered string ground state. **e** The distribution of string lengths for different lattice constants where the grey section covers those strings that are connecting only contiguous interior plaquettes and therefore are expected to have a different energy scale. We estimate the uncertainty in these values to be approximately 10–15%, based on the number of strings in the images. **f** The data from (**e**) plotted on a semi-logarithmic scale with exponential fits as described in the text.

Thermal annealing of the thicker islands is very efficient in reaching low energy states^6,27–29^, because it starts near the Curie point, at which the permalloy has reduced magnetization. Thus, the constraints predicated upon the binary nature of the island magnetization necessarily break down during this process. Figure 3a–c show typical real space snapshots obtained via MFM of annealed samples for different lattice constants, demonstrating the different sorts of string configurations observed. Figure 3d then plots the vertex statistics of annealed samples as a function of their lattice constants. For strongly coupled samples, i.e., small lattice constants, the data converge to the fractions expected for the disordered string ground state, where each string consists of three excited three-island vertices out of fourteen. The dashed lines represent the expected vertex fraction. Figure 3e, f show the distribution of string lengths from the MFM images, averaging over multiple images for each lattice constant. The observed exponential distribution is consistent with the strings being emergent thermal objects with weak mutual interactions. In the MFM images, they are frozen in place when the sample is cooled through the island moment blocking temperature. The greater fraction of longer strings for the larger lattice constants is an expected result of a higher effective temperature relative to the interaction strength.

**Fig. 4:**
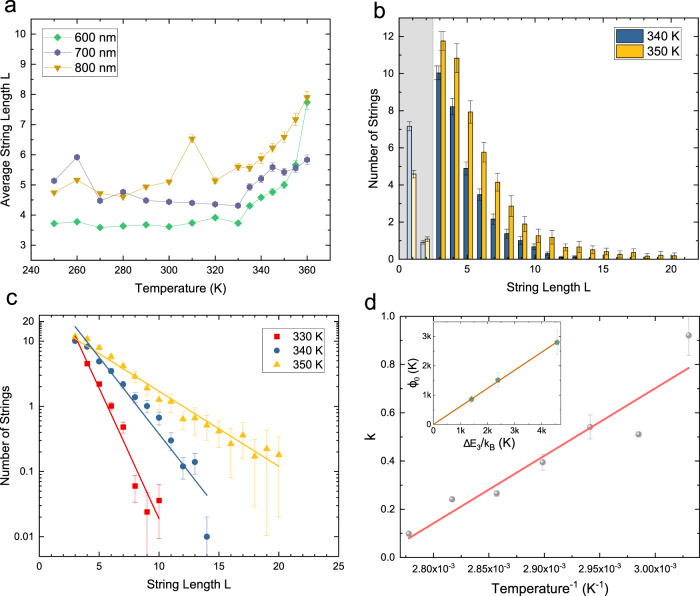
String statistics from XMCD-PEEM data. **a** The average length of strings as a function of temperature from the XMCD-PEEM data (measured in numbers of unhappy vertices). Note that the average string length is flat below *T* ≈ 330 K, indicating that the system falls out of thermal equilibrium. **b** The distribution of strings with various length for the *a* = 600 nm lattice, averaging over approximately 80 images (see Methods section). The grey section covers those strings that are connecting only contiguous interior plaquettes and therefore are expected to have a different energy scale. **c** The distribution of string length with a fit to a Boltzmann distribution *p*(*L*) $\propto$ e^−*Lk*^ as described in the text. The error bars in panels (**a**–**c**) represent the standard errors of the data taken at each temperature. **d** The fitted *k* from the Boltzmann distribution as a function of inverse temperature for *a* = 600 nm and 330 K ≤ *T* ≤ 360 K shows a linear dependence on the inverse temperature, *k*(*T*) = *ϕ*_*0*_/*T* − *ϕ*_1_. Inset: the fit parameter *ϕ*_*0*_ as a function of [Δ*E*_*3*_/*k*_*B*_] computed from micromagnetics for all three samples, where the error bars are the standard errors of the fit parameters.

In order to better probe the apparent activated behavior seen in Fig. 3e, f, we now consider the results of our XMCD-PEEM measurements. These data are taken on much thinner islands that can thermally reverse moment orientations on the time scale of the imaging. Figure 4a shows the average string length 〈*L*〉 taken from XMCD-PEEM data as a function of temperature for three different realizations of SFI with different lattice constants. At low temperature, the average length is only weakly temperature-dependent, suggesting that the system is trapped in a metastable state due to proximity to the superparamagnetic blocking temperature (see Supplementary Note 4 for more details regarding the moment configuration). In all three cases, the average string length increases substantially at the highest temperatures, indicating the onset of more extensive thermal fluctuations among the moments.

Because the energy of a string is proportional to its length, one expects from the Boltzmann distribution that the probability *p*(*L*) of finding a string of length *L* is exponential when the system is thermalized, as seen for the high temperature annealing data discussed above. In other words, we expect that *p*(*L*) $\propto$ e^−*Lk*^ when the moments are thermalized, where *k* is the reciprocal of a characteristic length and depends on temperature. Following a Boltzmann distribution, this corresponds to the average energy per unit length of the string, *ϕ*, divided by temperature, or *k* = *ϕ/T*.

We can relate these parameters to the microscopic behavior of the island moments. As strings at lower temperatures mostly connect *z* = 3 vertices, *ϕ* should be close to the energy difference between a Type I and a Type II vertex for *z* = 3, i.e., *ϕ* ≈ Δ*E*_*3*_*/*k_B_ = (*E*(II_3_) − *E*(I_3_))*/*k_B_. Since *ϕ* depends on the magnetization of the moments, and we know from previous measurements that the magnetization of thin permalloy films is temperature dependent in this regime^29^, *ϕ* must also be temperature dependent.

Figure 4b, c show the experimentally measured average distribution of string lengths, which is proportional to *p*(*L*), confirming the expected exponential Boltzmann behavior described above. To find that dependence, we extract the reciprocal characteristic length *k* as the slope of the linear fits in Fig. 4c for experiments at different temperatures. In Fig. 4d, we plot *k*(*T*) vs. 1/*T*, and we find a linear dependence, *k*(*T*) = *ϕ*_*0*_/*T* – *ϕ*_1._ This is equivalent to the energy cost per length, *ϕ*(*T*), having a linear temperature dependence of *ϕ*(*T*) = *ϕ*_*0*_ – *ϕ*_*1*_*T*. While the linear functional dependence of *ϕ*(*T*) cannot be easily justified over a wide temperature interval, a linear expression in our limited temperature range of measurement is a reasonable first order approximation.

Since we should be relatively close to the Curie temperature of permalloy for such thin islands^29^, we can validate our model by estimating the Curie temperature from *ϕ*(*T*). Because the magnetization goes to zero at the Curie temperature, then *ϕ(T)* must also, and we can therefore take *ϕ*(*T*_*Curie*_) = 0, yielding *T*_*Curie*_ ~ *ϕ*_*0*_/ *ϕ*_*1*_. We obtain from this estimate that *T*_*Curie*_ is of order 400 K for all three of our lattice constants (see Supplementary Table 3), quite consistent with experimental results for very thin permalloy films^29^.

We can further validate our fits from the magnitude of *ϕ*_*0*_. At low temperature, strings are composed primarily of *z* = 3 unhappy vertices, therefore *ϕ*_*0*_ must be close in magnitude to Δ*E*_*3*_*/*k_B_, which can be computed from micromagnetics. In the inset of Fig. 4d, we plot the fitted *ϕ*_*0*_ vs. Δ*E*_*3*_*/*k_B_, determined from a micromagnetic calculation (see Supplementary Table 3). We see that the quantities are proportional, and that (Δ*E*_*3*_*/*k_B_)/*ϕ*_*0*_ ~ 1.60. The deviation of the ratio from 1.0 is potentially attributable to limitations in the micromagnetic modelling (which, for example, does not include temperature dependence of the magnetization and interactions beyond the nearest neighbors), and to the fact that other types of vertex excitations also contribute to the string energy. The agreement is nonetheless striking in the sense that *ϕ*_*0*_ and *ϕ*_*1*_ are obtained not by fitting the data directly, but by fitting the temperature dependence of the Boltzmann parameter, *k*, which is itself derived from fits to data at each temperature. The results demonstrate that the physics of the topologically complex structure of SFI, with its large unit cell, can be robustly represented through the simple language of one-dimensional strings and their Boltzmann statistics.

## Discussion

The observed string correlations among unhappy vertices at low temperatures in SFI contrast sharply with the correlations among unhappy vertices in other vertex-frustrated systems. The correlations in the disordered portions of tetris ice are weak (i.e., exponentially decaying)^7,17^ while Shakti ice has algebraic correlations^6,8,17,30^, pointing to the criticality of its topological phase. In this case, the unhappy vertices in SFI are strongly correlated, forming one-dimensional collective emergent objects whose structure defines the magnetic moment ensemble, and whose motion and evolution define the overall kinetics of the system.

This string picture also has consequences for understanding the kinetics of the SFI system. As one-dimensional objects with ends fixed at the interior plaquettes, we can choose to consider the strings as defining a natural partition of the phase space into topological sectors. These sectors are then homotopy classes of the string configurations, i.e., ensembles of magnetic configurations corresponding to strings that are topologically equivalent, in that the strings can be continuously deformed without changing their ends. Therefore, topologically trivial kinetics correspond to bending and stretching modes of the strings that do not change the ends of the strings and thus do not alter the homotopy class. Relaxation and equilibration below the minimal energy of a sector requires the system to execute topologically nontrivial kinetics that can take it across homotopy classes. Such changes cut across homotopy classes, in the form of so-called string reconnection^20,22^, constituting a physical manifestation of one-dimensional “topological surgery” in which strings cross, disconnect^31,32^, and reconnect^33,34^, Similar phenomena are known theoretically to affect kinetics in some classical systems^35,36^, which evolve through rupture of the topological protection; they are likely to impact the kinetics here.

String correlations and their use in the partition of phase space into topological sectors are familiar in quantum topological matter, where they are a consequence of quantum entanglement^23,24,33,34^. Our results demonstrate that even in a classical system, frustration can impose correlations on a disordered state that are strong enough to generate a string phase. The robust string physics of SFI demonstrates that artificial spin ice systems provide a platform for such a framework in a purely classical context. Future examination of the string kinetics in SFI and related artificial spin ice systems should enable exploration of the physics of string excitations and their topological properties in a well-characterized and easily controlled experimental system.

## Methods

### Sample preparation

The artificial spin ice samples used in this work were fabricated through a process similar to that described in previous papers^6–8^. We first used electron-beam lithography to write patterns on Si/SiO_x_ substrates with spin-coated bilayer resists. Various thicknesses of permalloy (Ni_80_Fe_20_) films were then deposited into the patterns via ultrahigh vacuum electron beam evaporation, followed by aluminum capping layers that were 2 nm thick for XMCD-PEEM samples and that were 3 nm thick for MFM samples to mitigate oxidation of the underlying permalloy.

### Thermal annealing and MFM measurement

The samples used for thermal annealing and MFM measurements had lateral island dimensions of 220 nm × 80 nm and thickness of about 15 nm. These dimensions were chosen so that the magnetic moments of the nanoislands were frozen at room temperature. Five SFI arrays were designed with lattice constants of 300 nm, 320 nm, 360 nm, 440 nm, and 480 nm. All arrays were polarized along the [1,1] direction then heated to 818 K at a rate of 10 K/min. The samples were then held at 818 K for 15 min before being cooled to 673 K at a rate of 1 K/min and then cooled to room temperature for measurement^29^.

Two MFM scans were performed on each array to minimize location variances. Each MFM image contained about 900 total vertices and 1300 moments. Square ice lattices that were annealed simultaneously showed large ground state domains in MFM scans, demonstrating that the annealing protocols could successfully set vertices to the low energy states.

### XMCD-PEEM measurement

We conducted XMCD-PEEM experiments at the PEEM-3 endstation at beamline 11.0.1.1 of the Advanced Light Source, Lawrence Berkeley National Lab. The samples used for the XMCD-PEEM experiments had lattice constants of 600 nm, 700 nm, and 800 nm, and islands with approximate lateral dimensions of 470 nm × 170 nm. The exact dimensions measured by SEM are given in Supplementary Table 2. The permalloy thicknesses of PEEM samples were approximately 2.5 nm, and the island moments were thermally active in the tested temperature window. We heated the sample from 250 K to 360 K in 5–10 K steps and took 100 PEEM images at the Fe L_3_ absorption edge at each temperature point. The 100 PEEM images consisted of ten exposures with a left-circularly polarized X-ray beam followed by ten exposures with a right-circularly polarized beam, repeated five times. Charging problems during the experiment caused a small fraction (≤20%) of the images to be out of focus, and these images were excluded from the analysis. The exposure time was set to 0.5 seconds and the total acquisition time at each temperature was about 150 s, including computer read-out between exposures. The PEEM image field of view was set at 15 ×15 to 18 ×18 µm^2^ and there were about 500 islands within each image. We used code prepared with MATLAB to extract the intensity for each island from every PEEM image. The intensity values usually fall into two groups, one with higher average and the other with lower average. We can then resolve the moment direction for each single island for each image. The island flip rate was obtained by calculating the fraction of islands that changed their moment directions between two sequential PEEM images with the same X-ray polarity taken at each temperature, divided by the acquisition time of two images. In addition to the data shown in Fig. 4, additional PEEM measurements and detailed associated parameters are discussed in Supplementary Note 4.

## Supplementary information

Supplementary Information

## Data Availability

Experimental and simulation data generated in this study have been deposited in the Dryad database under 10.5061/dryad.jdfn2z3c2^37^
