# Supplementary material for: String Phase in an Artificial Spin Ice
Source: Nat Commun. 2021 Nov 11;12:6514. doi: 10.1038/s41467-021-26734-6 (PMC8585881; doi:10.1038/s41467-021-26734-6)
Supplement: Supplementary file 1 — Supplementary Information [file 41467_2021_26734_MOESM1_ESM.pdf]

Supplementary Information

String Phase in an Artificial Spin Ice

Xiaoyu Zhang[1,2,3], Ayhan Duzgun[4], Yuyang Lao[2,3], Shayaan Subzwari[1], Nicholas S. Bingham[1], Joseph Sklenar[2,3,5], Hilal Saglam[1], Justin Ramberger[6], Joseph T. Batley[6], Justin D. Watts[6,7] Daniel Bromley[8], Rajesh V. Chopdekar[9], Liam O'Brien[8], Chris Leighton[6], Cristiano Nisoli[4], & Peter Schiffer[1,2,3,10]

[1]*Department of Applied Physics, Yale University, New Haven, CT 06511, USA*

[2]*Department of Physics, University of Illinois at Urbana-Champaign, Urbana, Illinois 61801, USA*

[3]*Frederick Seitz Materials Research Laboratory, University of Illinois at Urbana-Champaign, Urbana, Illinois 61801, USA*

[4]*Theoretical Division and Center for Nonlinear Studies, MS B258, Los Alamos National Laboratory, Los Alamos, New Mexico 87545, USA*

[5]*Department of Physics and Astronomy, Wayne State University, Detroit, Michigan 48201, USA*

[6]*Department of Chemical Engineering and Materials Science, University of Minnesota, Minneapolis, Minnesota 55455, USA*

[7]*School of Physics and Astronomy, University of Minnesota, Minneapolis, Minnesota 55455, USA*

[8]*Department of Physics, University of Liverpool, Liverpool L69 3BX, United Kingdom*

[9]*Advanced Light Source, Lawrence Berkeley National Laboratory, Berkeley, CA 94720, USA*

[10]*Department of Physics, Yale University, New Haven, CT 06511, USA*

Supplementary Note 1: Monte Carlo simulations

In order to better understand the Santa Fe Ice system, we have run extensive Monte Carlo simulations. We considered a system with 4 x 8 unit cells of the lattice (the unit cell is shown in Figure 1a in the main text), which consists of 8 x 8 composite cells or 1536 spins. We impose periodic boundary conditions and calculate the system energy using a vertex model where only nearest-neighbor interactions between collinear and perpendicular islands are included (*Supplementary Fig. 1*). The two spin interaction energies, $\epsilon_{\perp}$ and $\epsilon_{\parallel}$ correspond to the coupling between moments that are converging in the vertex perpendicular and parallel to each other respectively and are defined in *Supplementary Fig. 1*. In other words, in this approximation, the spins only interact with other spins that share a common vertex. The value of $\epsilon_{\parallel}$ defines an energy (and thus temperature) scale which naturally depends on the details of the island geometry and spacing in a real system.

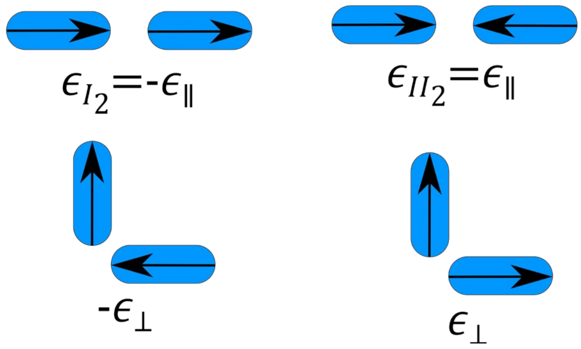

Supplementary Fig. 1. Schematics defining the couplings in the Metropolis Monte Carlo simulations.

The value of $\frac{\epsilon_\perp}{\epsilon_\parallel}$ determines the ground state of the system as shown in *Supplementary Fig. 2*. The two possible ground states are either a long-range-ordered state or a disordered string state with strings all of length three excitations. Note that, even in the case of a long-range antiferromagnetic ground state, the excitation profile is made of strings, due to the topological constraints for the number of unhappy vertices that can connect to a given plaquette. In *Supplementary Table 1*, we show the predicted population statistics of vertices at high temperature and zero temperature. Values at high temperature are merely the relative counts of the vertex type for a random distribution of moments. Values at zero temperature are different for the two different possible ground states. For the disordered string ground state, vertices of coordination two and four are all in their lowest energy configuration (Type I_2 and Type I_4), whereas 3/14 of the z = 3 vertices in a composite cell are unhappy vertices, i.e., in the Type II_3 moment configuration. For the long-range-ordered state, vertices of coordination *z* = 3 and *z* = 4 are all in their lowest energy configuration (Type I_3 and Type I_4), whereas all of the *z* = 2 vertices are unhappy vertices, i.e., in the Type II_2 moment configuration. As a full long-range-ordered state was not seen in the measurements reported in this work, we limit ourselves to simulations for ratios below the threshold that would lead to long range order.

Vertex type	High temperature state	Disordered string ground state vertex fractions	Long-range-ordered ground state vertex fractions
I_4	≈ 2/16 = 0.125	1	1
II_4	≈ 4/16 = 0.25	0	0
III_4	≈ 8/16 = 0.5	0	0
IV_4	≈ 2/16 = 0.125	0	0
I_3	≈ 2/8 = 0.25	11/14 ≈ 0.79	1
II_3	≈ 4/8 = 0.5	3/14 ≈ 0.21	0
III_3	≈ 2/8 = 0.25	0	0
I_2	≈ 2/4 = 0.5	1	0
II_2	≈ 2/4 = 0.5	0	1

Supplementary Table 1. Predicted vertex fractions at high temperature and in the two ground states (i.e., at zero temperature).

For the simulations, the system was cooled through 500 steps of exponentially decaying temperature values which approximately corresponds to setting $T(i+1) = 0.99\ T(i)$ for i^{th} and $i+1^{th}$ steps. At each temperature step, 300,000 trials per spin were performed. The system energy was recorded after each of the last 60% of these trials (180,000 per spin), and these were used to calculate the heat capacity. The average of the vertex counts over the duration of all of the 300,000 updates at each temperature was used to calculate the relative populations.

The simulation procedure is as follows. We start with very high temperature and slowly anneal to zero temperature using a standard Metropolis algorithm, which uses single spin flips that mimic the thermal evolution seen in the PEEM experiments. We

collect statistics of vertex populations, and we compute the heat capacity and the entropy during the annealing process. The heat capacity C_v per spin is calculated from thermal fluctuations using the equation

$$\frac{C_v}{k_B} = \frac{1}{(k_B T)^2} \frac{\sigma^2(E_N)}{N} \tag{1}$$

where E_N is the total energy of the system which has N spins, and σ is the standard deviation of E_N. The entropy per spin is calculated by

$$\frac{S(T)}{k_B} = \ln 2 + \int_{\infty}^{T} \frac{C_v}{k_B} \frac{dT}{T} \quad . \tag{2}$$

Supplementary Fig. 3 – Fig. 5 show snapshots for $\frac{\epsilon_\perp}{\epsilon_\parallel} = 1.3$. For these interactions, within the vertex approximation, the ground state corresponds to the disordered string ground state. The figures show a relatively high temperature state, $k_B T/\epsilon_\parallel = 1.35$, an intermediate temperature state, $k_B T/\epsilon_\parallel = 0.40$, and a configuration near T = 0. As the system is cooled, the high density of strings reduces to the disordered string ground state, where strings start and end at nearest interior plaquettes. At sufficiently low temperature, essentially all of the z = 4 vertices are of Type I_4 due to its low energy.

Supplementary Fig. 6 shows the corresponding vertex statistics of z = 4, z = 3, and z = 2 vertex types and the curve for entropy as the system is cooled. In the high temperature limit, populations are entropic, i.e., proportional to their relative multiplicity, as also listed in *Supplementary Table 1*. Note the two bumps in the specific heat. The one at larger temperature denotes the crossover into a regime where the vertices obey

the ice rule. The one at lower temperature denotes the crossover to the disordered string ground state. Note also the residual entropy at zero temperature demonstrating the extensive degeneracy of the ground state. Finally, note that the population of unhappy vertices, or Type II_3, do not tend to zero as temperature is lowered, because they form the strings. Naturally, as for the case of pyrochlore ice and honeycomb ice, the dipolar interaction, here neglected, might induce ordering at very low temperature.

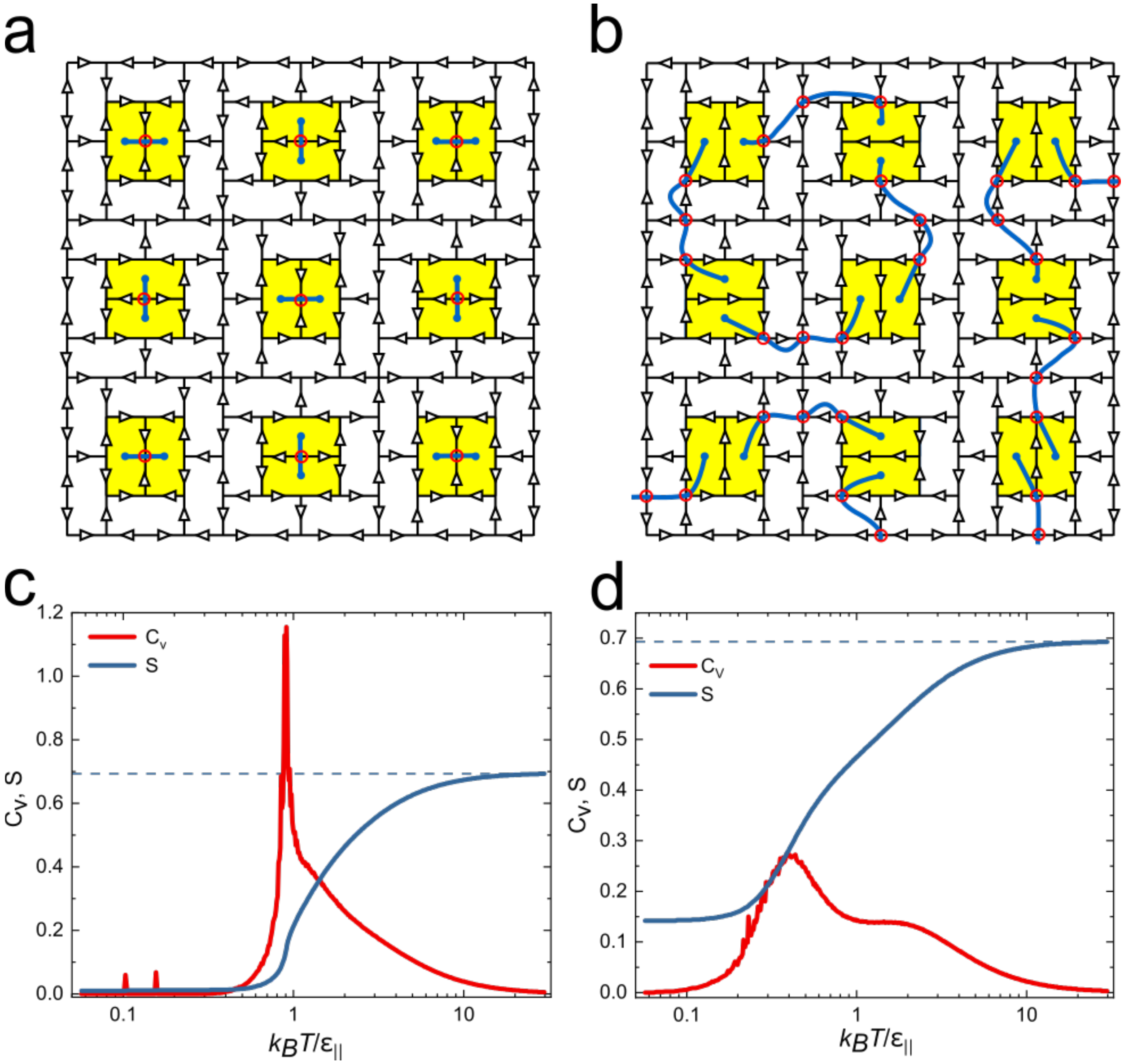

Supplementary Fig. 2: Santa Fe ice ground states. Schematic of the moment configurations and the strings, showing (a) the long-range-ordered ground state, and (b) the disordered string ground state. (c) and (d) specific heat (C_V) and entropy (S) per moment as a function of effective temperature from Monte Carlo calculations for $\frac{\epsilon_\perp}{\epsilon_\parallel}$= 1.9 and 1.3, respectively. The simulations yield the long-range-ordered state and the disordered string ground state in these two cases. Note the sharp peak associated with the transition to long range order, the broad peak associated with the evolution to the disordered ground state, and the shoulder in each case associated with the development of short-range order on the vertices (the small low-temperature peaks are noise in the simulation). Note the residual entropy in the entropy curve for the disordered state. Entropy at infinite temperature is ln2 (dashed blue line).

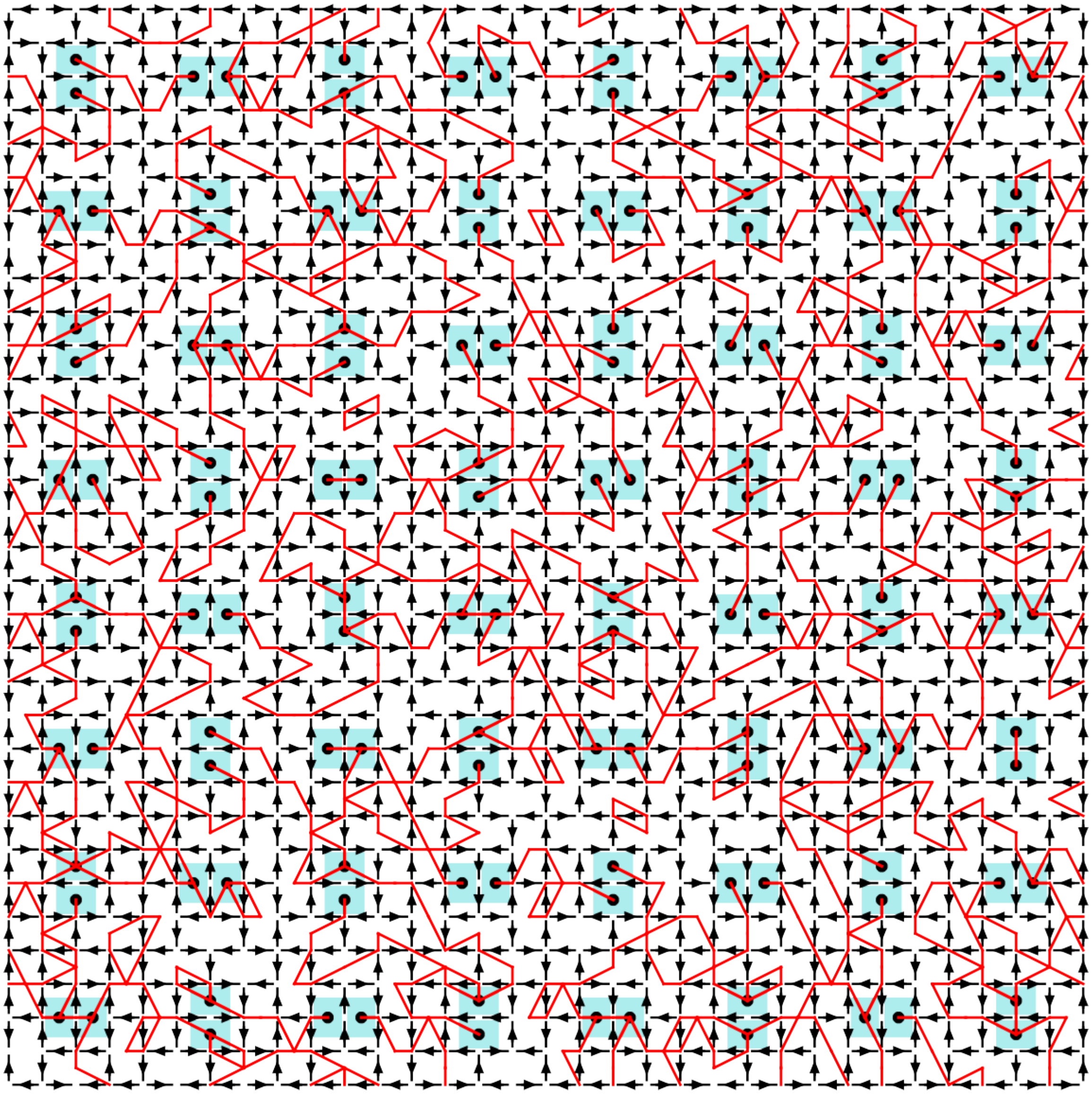

Supplementary Fig. 3. Snapshots from Monte Carlo simulation for $k_B T/\epsilon_\parallel$ =1.35 and $\frac{\epsilon_\perp}{\epsilon_\parallel}$= 1.3. Red lines show the strings of unhappy vertices, and the full system size of 8 × 8 composite cells is shown. Note that, at this relatively high temperature, the high density of excitations leads to a higher density of strings and the presence of longer strings and loops.

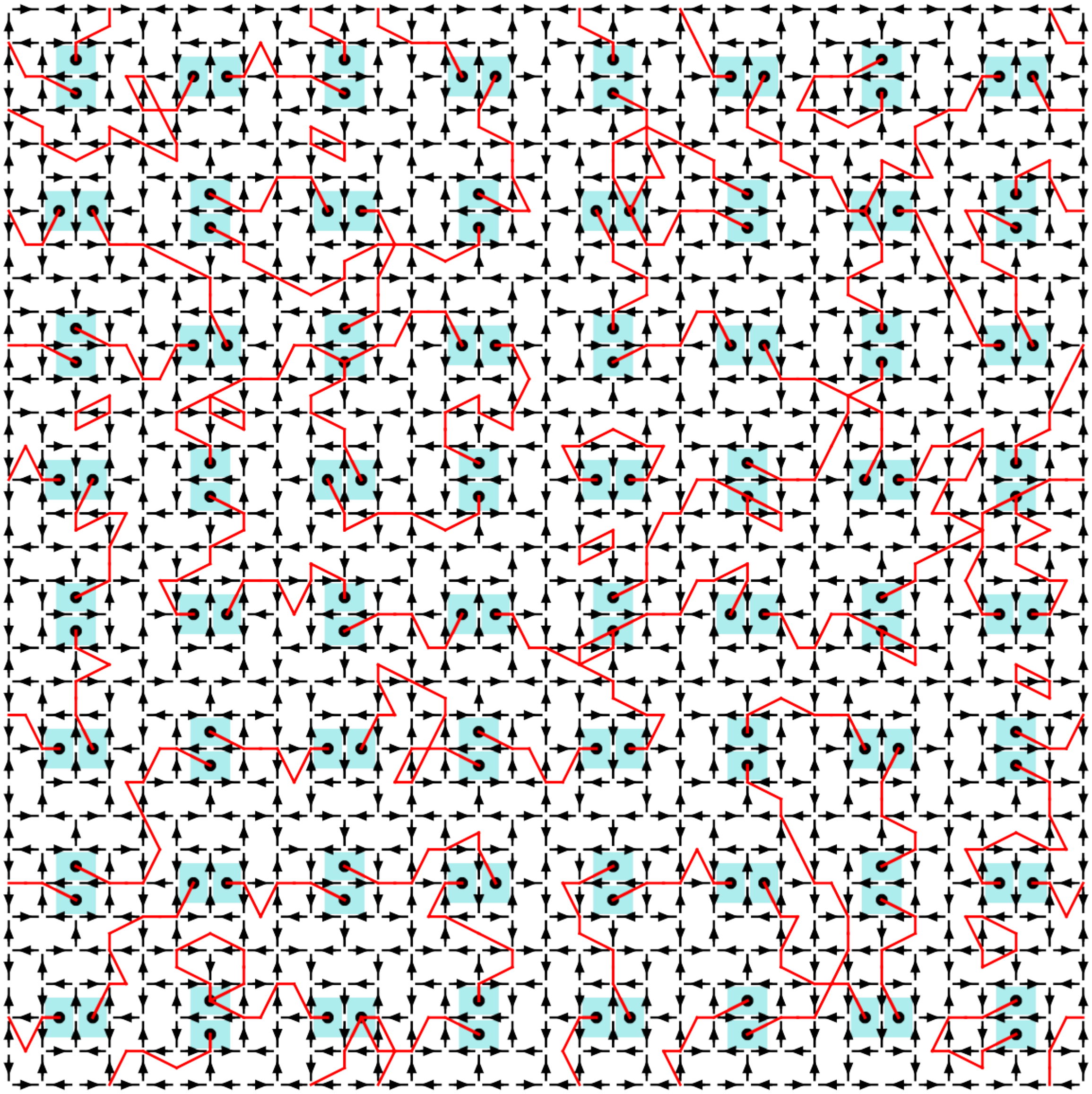

Supplementary Fig. 4. Snapshots from Monte Carlo simulation for $k_B T/\epsilon_\parallel$= 0.40 and $\frac{\epsilon_\perp}{\epsilon_\parallel}$= 1.3. Red lines show the strings of unhappy vertices, and the full system size of 8 × 8 composite cells is shown. The picture shows an excited string state where strings are generally longer than the three unhappy vertices predicted in the ground state and can run between interior plaquettes farther away.

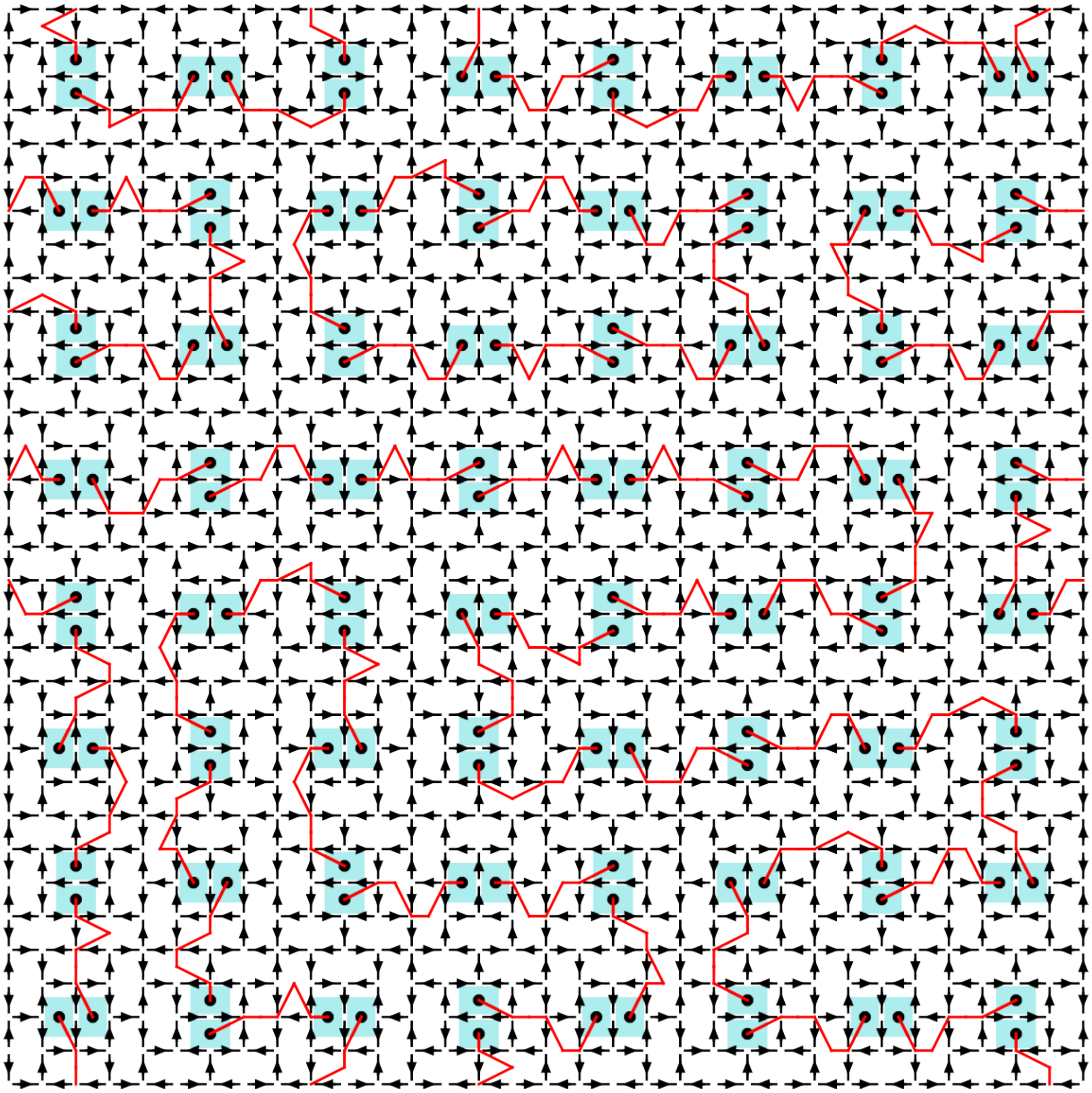

Supplementary Fig. 5. Snapshots from Monte Carlo simulation for $k_B T/\epsilon_\parallel$ = 0.05 and $\frac{\epsilon_\perp}{\epsilon_\parallel}$= 1.3. Red lines show the strings of unhappy vertices, and the full system size of 8 × 8 composite cells is shown. The system here is in the disordered string ground state.

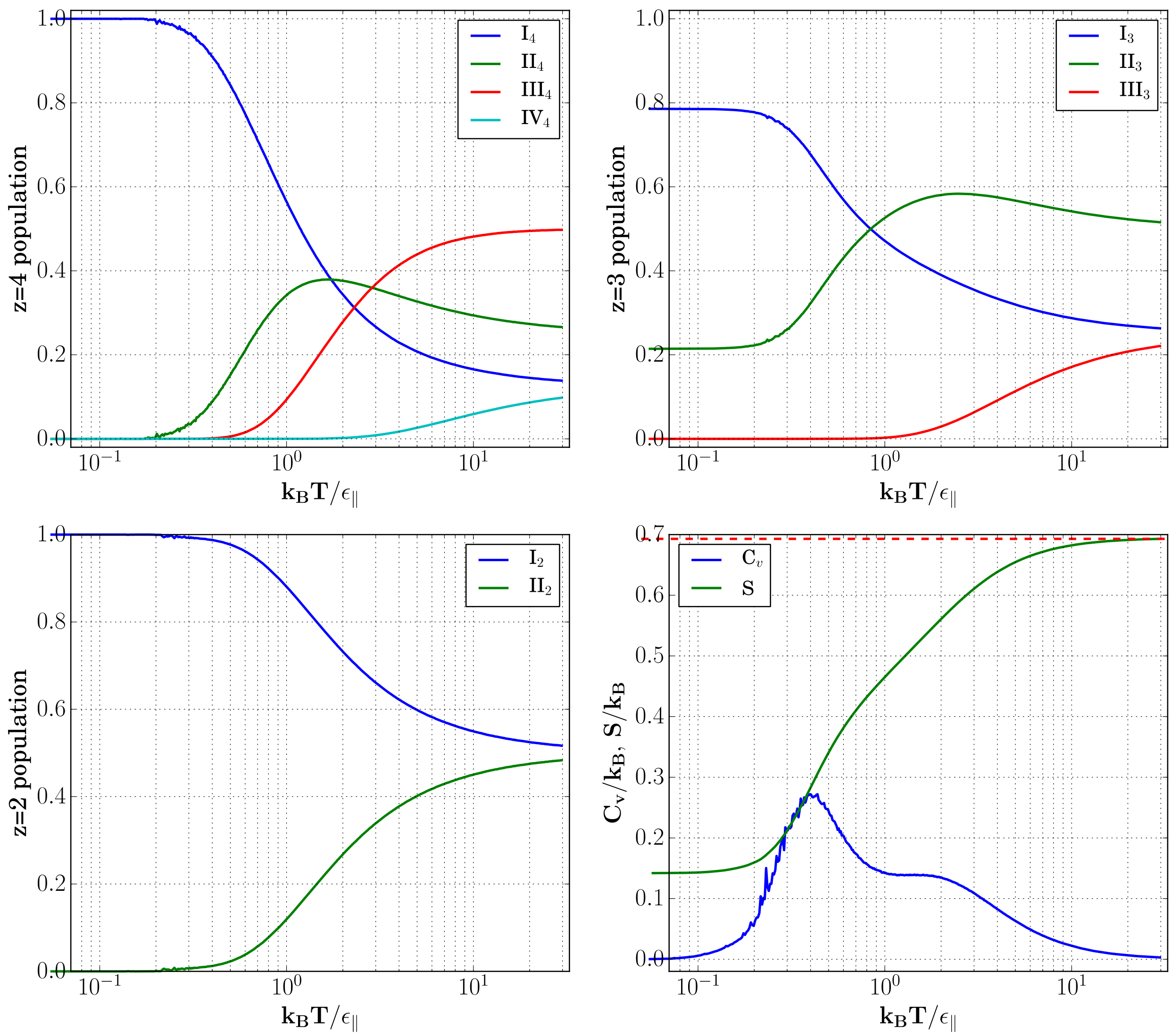

Supplementary Fig. 6. Simulated vertex statistics for $\frac{\epsilon_\perp}{\epsilon_\parallel}$= 1.3. Relative counts of vertices for coordination numbers z = 4, z = 3, z = 2 as well as entropy and specific heat are shown (same as for Supplementary Fig. 2, included here for easy comparison). Note that all vertex types tend to their ground states except z=3 vertices: the unhappy vertices, or Type II_3, do not tend to zero as temperature is lowered, because they form the strings. The two bumps in the specific heat signal onset of the ice-rule, and then of the string state. Note also the residual entropy at T=0. Dashed red line in the entropy graph indicates ln 2.

Supplementary Note 2: Peripheral and Interior Plaquettes: Numbers of Connected Unhappy Vertices

Santa Fe Ice has multiple coordination numbers at the different vertices, in that vertices can have two, three and four islands, or coordination numbers of $z = 2$, 3, and 4. Within the SFI structure, the interior and peripheral plaquettes differ in that the interior plaquette has a $z = 2$ vertex, while the peripheral plaquette vertices are $z = 3$ or 4. This can be readily seen in *Supplementary Fig. 7*.

For the interior plaquettes, to enable each of the $z = 3$ vertices to be "happy", i.e., in its vertex ground state, the $z = 2$ vertex must be in its excited "unhappy" state of the two moments in opposite directions, as can be readily seen in *Supplementary Fig. 7 b*. Flipping one of the moments in the $z = 2$ vertex to put it into its ground state would simply make one of the $z = 3$ vertices "unhappy". The structure thus requires that the interior plaquettes be connected by at least one unhappy vertex around its edges, although it is easy to see that reversing other moments could produce any odd number of unhappy vertices, e.g., either one or three or five. Those plaquettes are therefore intrinsically frustrated in that they cannot have all vertices in a ground state, and their frustration affects nearby, unfrustrated peripheral plaquettes.

For the peripheral plaquettes, it is possible to put all of the vertices around the plaquette in their lowest energy "happy" state, as demonstrated in *Supplementary Fig.7 c* and d. By flipping any of the moments, one would create a pair of unhappy vertices on either side of that moment, and one could create either four or six unhappy vertices by strategic choice of which moments to flip. Therefore, the peripheral plaquettes must be connected by an even number of unhappy vertices (including possibly zero vertices as

shown in the figure). These two conditions lead naturally to the string construction described in the main text

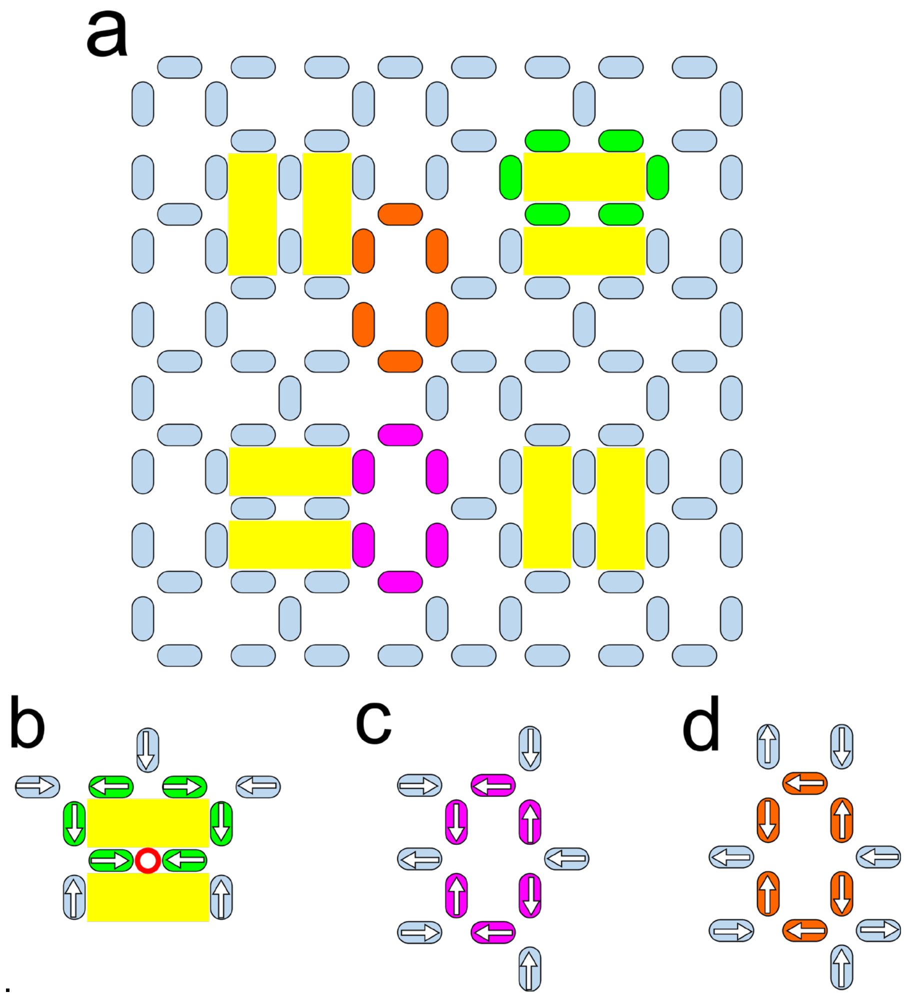

Supplementary Fig. 7. Santa Fe Ice structure and sample plaquettes. (a) A full unit cell of the SFI structure with interior and peripheral plaquettes highlighted in green (interior) and pink and orange (peripheral). (b) The sample interior plaquette with moment directions indicated. There is a single unhappy vertex associated with the z = 2 vertex, emphasized with a red circle. Flipping a moment around the edges of the plaquette would simply place one of the z = 3 vertices into an unhappy state or add a pair of unhappy vertices – therefore the plaquette must have an odd number of unhappy vertices. (c,d) Sample peripheral plaquettes with moment directions indicated. The two plaquettes have all z = 3 vertices (for c), or a single z = 4 vertex (for d). In both cases the moments can be assigned as shown, so that all of the vertices are in their ground state, i.e., all are happy. Flipping any moment around the plaquette would result in a pair of unhappy vertices, and therefore these plaquettes must have an even number of unhappy vertices (including zero as a possible even number).

Supplementary Note 3: Additional MFM data

Below we provide sample MFM images after high-temperature annealing (details of annealing provided in the Methods section). We repeated the annealing experiment two times on two different samples. To minimize the sample variance and location variance, we took two MFM images at different locations on each array after each annealing run and calculated the vertex fraction, shown in Figure 3 in the main text. *Supplementary Fig. 8* gives examples of MFM images of SFI arrays with different lattice constants.

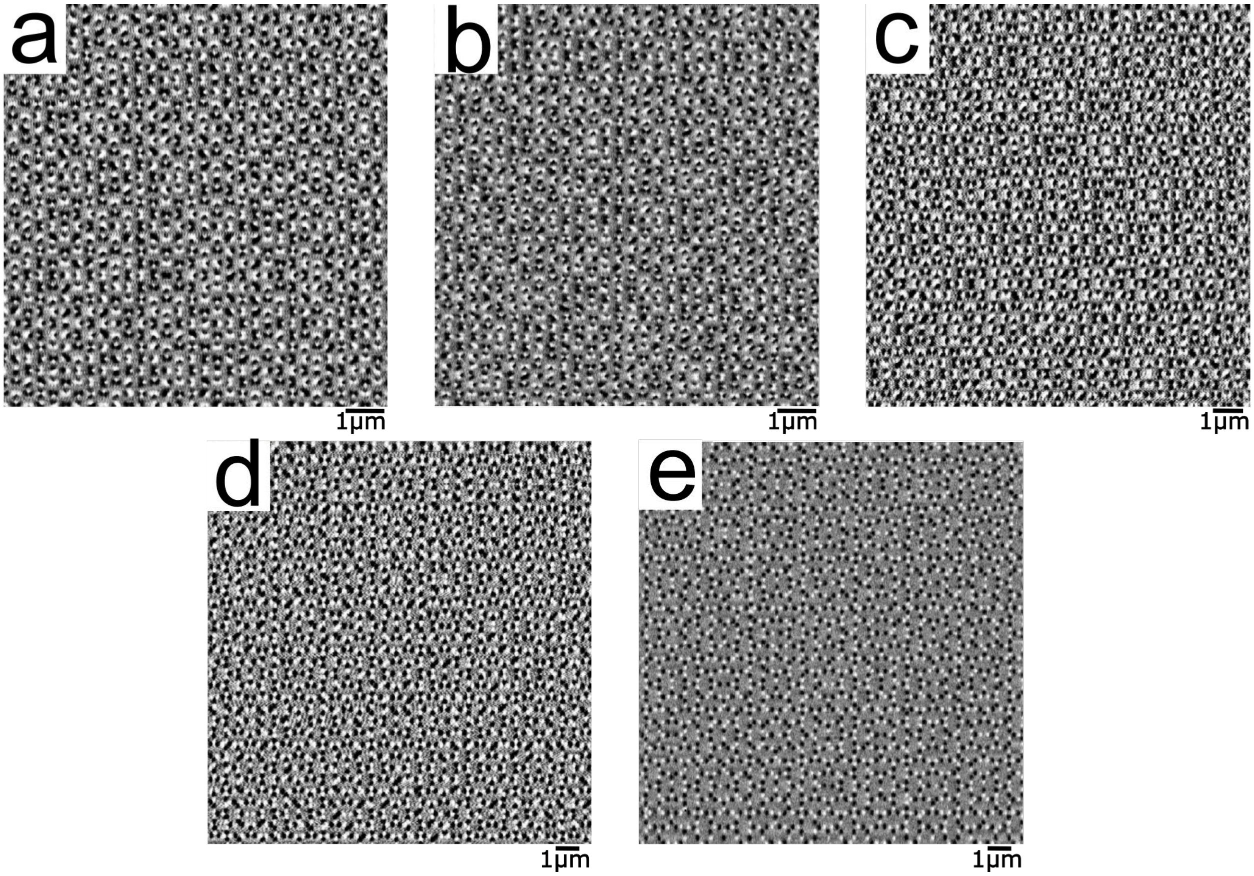

Supplementary Fig. 8. MFM images of SFI lattice with lattice constant a) 300 nm, b) 320 nm, c) 360 nm, d) 440 nm, e) 480 nm after annealing at 818 K for 15 mins.

Supplementary Note 4: Additional PEEM data

We performed five different runs of PEEM measurements on SFI, with different samples in each case. The exact island dimensions for each run are shown in *Supplementary Table 2* For the first, second, fourth, and fifth PEEM runs, we focused on dynamics, taking a series of 100 PEEM images at each temperature as samples were stepwise heated or cooled. The 100 PEEM images consisted of ten exposures at the Fe L_3 edge with a left-circularly polarized X-ray beam followed by ten exposures with a right-circularly polarized beam, repeated five times. The exposure time was set to 0.2 to 0.5 seconds and the total acquisition time at each temperature was about 130 to 150 seconds including computer read-out between exposures. The PEEM images were analyzed as described in the Methods section in the main text. For the third PEEM run, we took two PEEM-XMCD images at 10 different locations at each temperature point to obtain better statistics. Each image was constructed from four PEEM images with a left-polarized X-ray beam and four with a right-polarized beam; we took the average of every four PEEM images and subtracted the averaged images with left-polarized X-rays from the right-polarized X-rays to obtain a PEEM-XMCD image. The exposure time for each PEEM image was 0.7 seconds for better intensity statistics per image.

We did not observe significant temperature dependence in the moment configuration from the first four PEEM experiments. Presumably the moments were trapped in metastable states for those samples in our temperature range, due to the topological complexity of the lattice combined with structural disorder associated with the lithography. The detailed results for these experiments are therefore not included in this paper, but they are available upon request. The data in the remainder of this section and

in the main text is from the fifth run, where significant temperature dependence was observed at the upper end of the temperature range measured, enabling us to study the thermal properties of the strings. The results in the remainder of this section focus on data from the fifth run.

PEEM Experiments	600 nm SF array		700 nm SF array		800 nm SF array	
	Length (nm)	Width(nm)	Length (nm)	Width(nm)	Length (nm)	Width(nm)
First	430±3.9	143±6.2	455±2.8	160±2.6	484±4.3	175±2.2
Second	439±6.5	146±6.5	448±5.2	156±2.2	471±1.2	169±3.2
Third	455±5.2	173±2.1	484±4.5	192±2.4	498±1.7	193±1.6
Fourth	470±3.6	191±8.6	501±3.7	205±5.2	511±6.3	210±2.6
Fifth	482±1.5	189±1.5	477±4.9	186±3.8	477±1.2	179±1.5

Supplementary Table 2. Island dimensions for all five PEEM runs.

We calculated the vertex statistics and island flip rate as shown in *Supplementary Fig. 9*. *Supplementary Fig. 10* shows the plot of k vs. $1/T$ with a linear fitting for (a) 700 nm and (b) 800nm. The ϕ_0, the slope of k vs. $1/T$, is compatible with $\Delta E_3/k_B$, where the exact numbers are given in *Supplementary Table 3*.

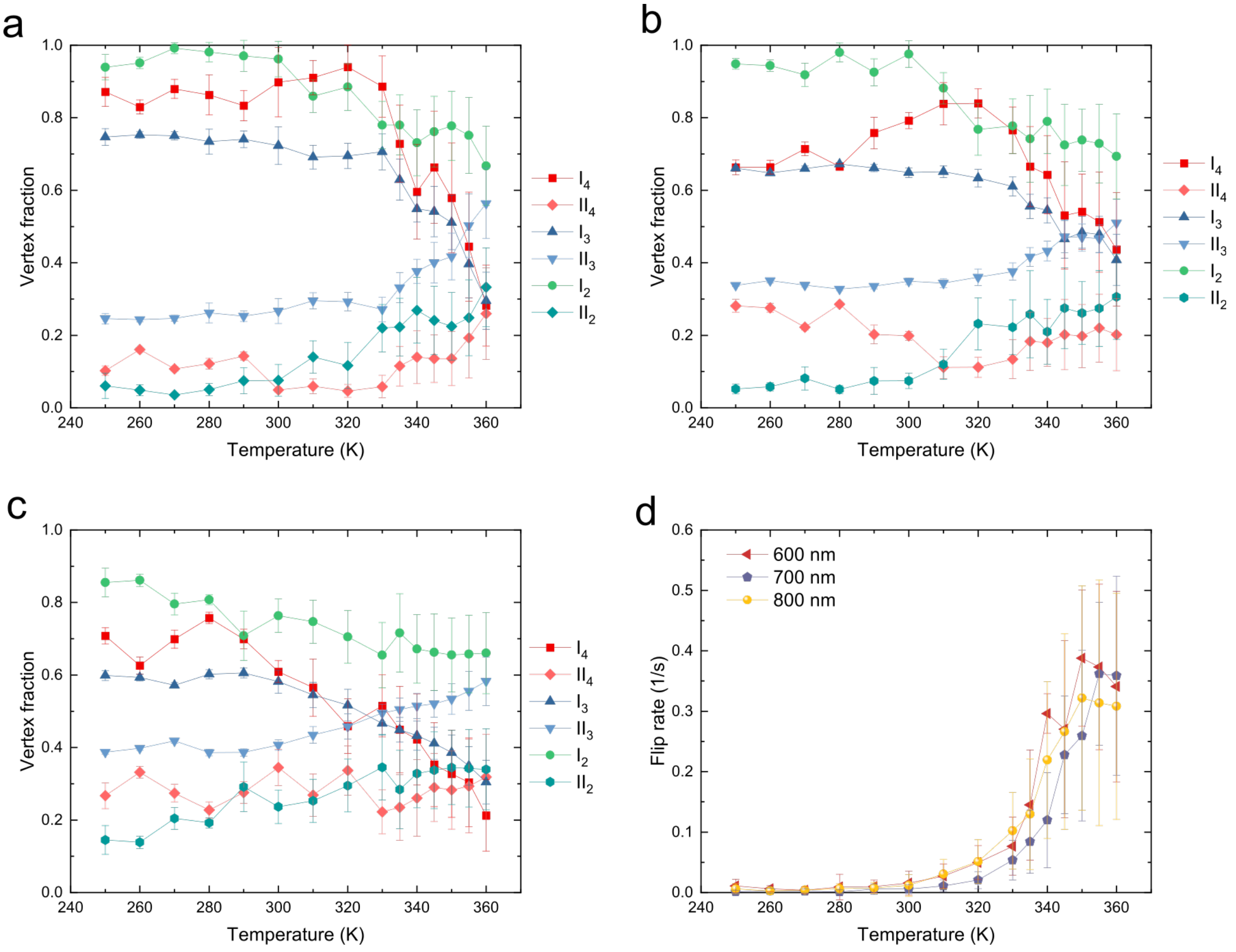

Supplementary Fig. 9. Vertex statistics for Type I vertices and Type II vertices for vertex coordination of four, three, and two, colored in red, blue, and green, respectively for (a) 600 nm, (b) 700 nm, and (c) 800 nm SFI. (d) Average islands flip rate as a function of temperature for a= 600 nm (red), 700 nm (violet), and 800 nm (yellow) SFI. The error bars represent the standard deviations of the data.

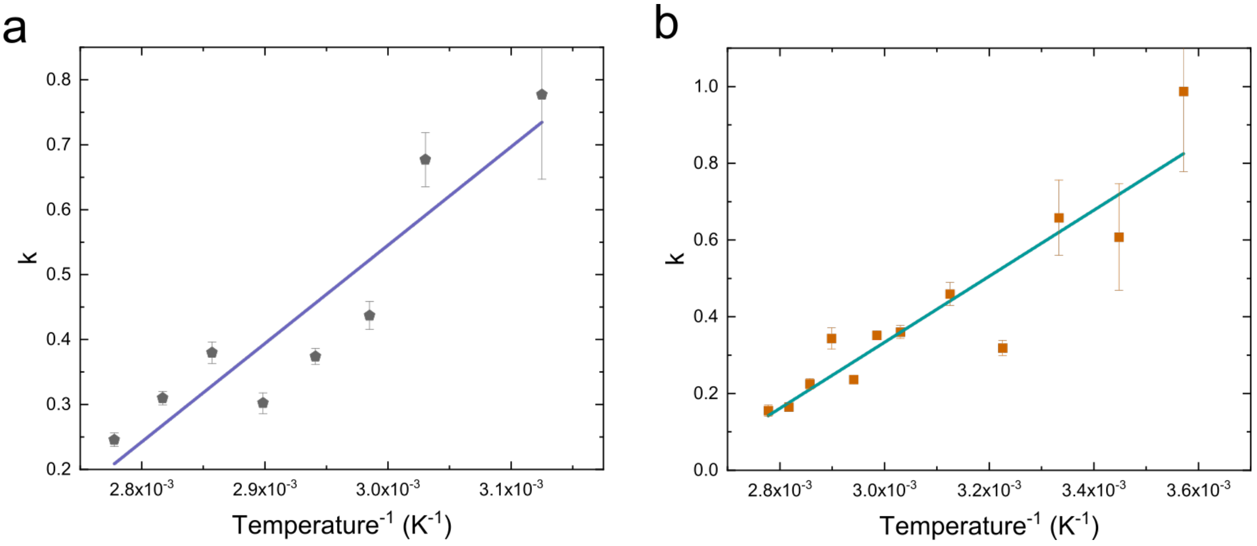

Supplementary Fig. 10, The string tension fit from the Boltzmann distribution as a function of inverse temperature for *(a) 700 nm for 320 K ≤ T ≤ 360 K and (b) 800 nm SFI for 280 K ≤ T ≤ 360.* The error bars are the standard errors of the fit parameters.

SFI array	E_{I2} (E-18 J)	E_{II2} (E-18 J)	E_{I3} (E-18 J)	E_{II3} (E-18 J)	$\Delta E_3/k_B$ (1000 K)	ϕ_0 (100 K)	ϕ_1	ϕ_0/ϕ_1 (K)
600 nm	1.42	1.55	2.00	2.06	4.57	27.98	7.69	364
700 nm	1.41	1.47	2.07	2.10	2.39	15.14	4.00	378
800 nm	1.39	1.42	2.05	2.07	1.41	8.60	2.25	382

Supplementary Table 3. Magnetostatic energies for the ground state and the first excited state of Z = 2 and 3 vertices, calculated by micromagnetic simulation program MUMAX3 [1], using the island dimensions for the fifth PEEM run. Also shown are the fit parameters ϕ_0 and ϕ_1. As described in the text, ϕ_0/ ϕ_1 gives an approximate value of the Curie temperature of the permalloy films.

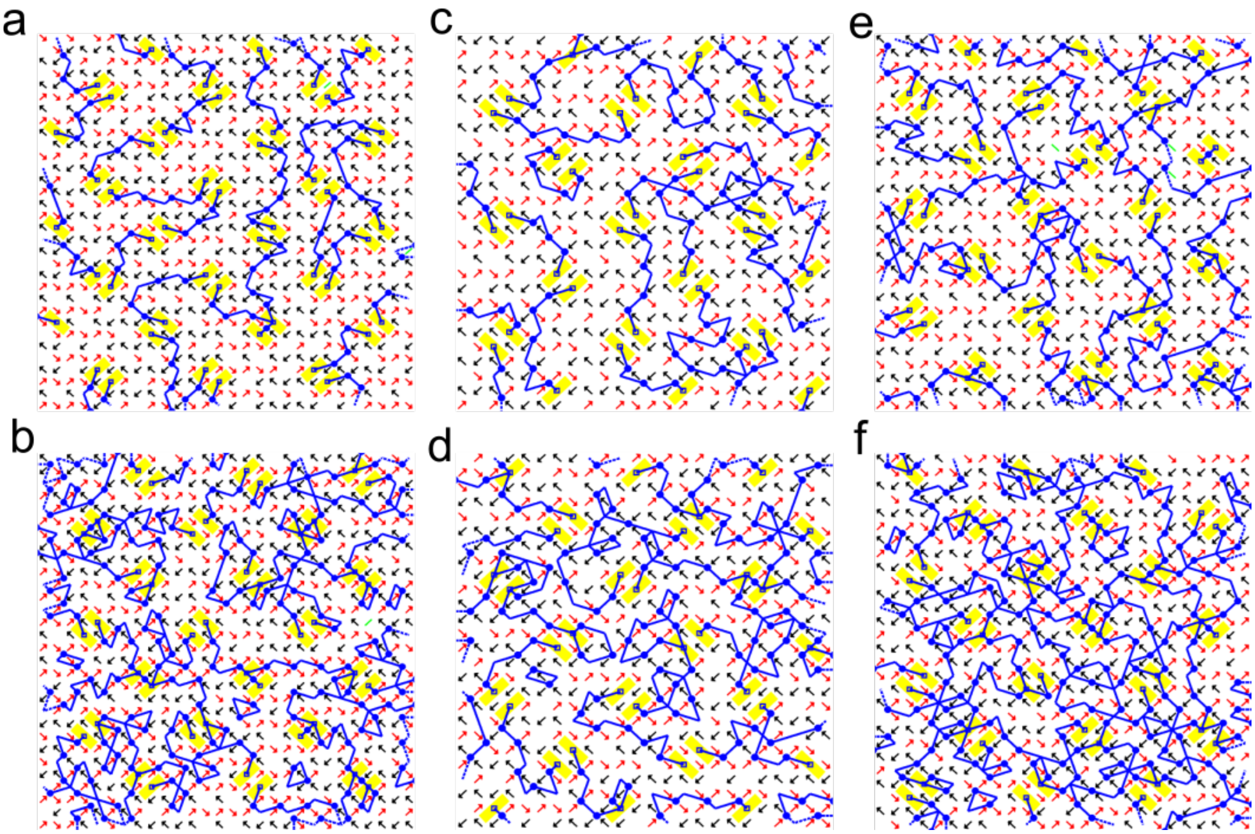

Supplementary Fig. 11. Examples of digitized PEEM images with both the moments and strings represented for SFI of a = 600 nm at (a) 250 K and (b) 350 K, 700 nm at (c) 250 K and (d) 350 K, and 800 nm at (e) 250 K and (f) 350 K. Broken lattice lines indicate that the islands were outside of the PEEM image boundary. Green lines indicate that the moment directions were not resolvable from PEEM images. Dashed blue lines give the possible string configurations around missing moments.

Supplementary Note 5: Calculation of String Length Distribution.

We use a simple graph theory approach to calculate the string length distribution from our experimental maps of the moments acquired from PEEM and MFM measurements. We created an adjacency matrix $\boldsymbol{A}$ for each PEEM image in which each row or column index corresponds to a vertex in the PEEM image. If the ith vertex and the jth vertex are connected by a string, the a_{ij} and a_{ji} entries in the matrix $\boldsymbol{A}$ are 1. Otherwise, the entries are 0. An example is shown in *Supplementary Fig. 12*. If $a_{ij}^{(L)}$ is a non-zero number in the matrix $\boldsymbol{A}^{L}$, the ith vertex and the jth vertex are connected by a string of length L. We calculate the matrix $\boldsymbol{A}^{L}$ for integral L = 1 to N where N is the maximum string length. The number of strings of length L comes from the number of entries $a_{ij}^{(L)} > 0$ ($i<j$). Note that $a_{ij}^{(L)}$ must satisfy three conditions: (1) both the ith vertex and the jth vertex have a string that directly go into an interior plaquette, (2) $a_{ij}^{(m)} = 0$ for all $m<L$, and (3) there is no such index t (the tth vertex is also directly connect to an interior plaquette), where $a_{it}^{(p)} > 0$, $a_{tj}^{(q)} > 0$, and $p+q=L$. The first condition guarantees that we only look at the strings connecting interior plaquettes. The second condition makes sure every segment on a string is counted only once. And the third condition excludes the strings that go from interior plaquettes A to B through C.

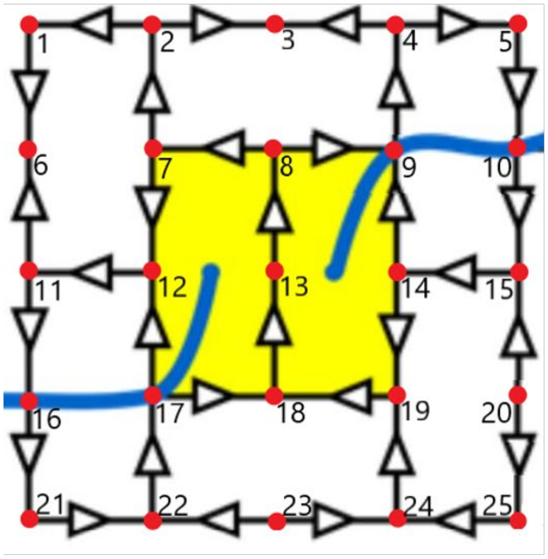

	1	2	…	9	10	…	16	17	…	25
1	0	0	…	0	0	…	0	0	…	0
2	0	0	…	0	0	…	0	0	…	0
…	0	0	…	0	0	…	0	0	…	0
9	0	0	…	0	1	…	0	0	…	0
10	0	0	…	1	0	…	0	0	…	0
…	0	0	…	0	0	…	0	0	…	0
16	0	0	…	0	0	…	0	1	…	0
17	0	0	…	0	0	…	1	0	…	0
…	0	0	…	0	0	…	0	0	…	0
25	0	0	…	0	0	…	0	0	…	0

Adjacency matrix A

*Supplementary Fig. 12. An example of a spin map and its adjacency matrix **A**. The 9th and 10th vertices and 16th and 17th vertices are connected by strings. Therefore, the $a_{9,10}$, $a_{10,9}$, $a_{16,17}$, and $a_{17,16}$ are 1, marked in red. Other entries are 0.*

SUPPLEMENTARY REFERENCES

[1] Vansteenkiste, A., Leliaert, J., Dvornik, M., Helsen, M., Garcia-Sanches, F. & Waeyenberge, B. V. The design and verification of mumax3, *AIP Advances* **4**, 107113 (2014).
